# Health problems among Forcibly Displaced Myanmar Nationals (FDMNs) admitted to the Medicine ward of Cox's Bazar Medical College Hospital

**DOI:** 10.1016/j.jmh.2022.100123

**Published:** 2022-05-31

**Authors:** Mohammad Ismail, Mohammad Farhad Hussain, Mohammad Abdullah al Hasan, AHM Mustafa Kamal, Monjur Rahman, Mohammad Jahid Hasan

**Affiliations:** a Department of Biochemistry, Dhaka Medical College Hospital, Dhaka 1000, Bangladesh; b Department of Medicine, Cox's Bazar Medical College, Cox's Bazar, Bangladesh; c OSD, Directorate General of Health Services, Bangladesh; d Department of Intensive Care Unit, Kurmitola General Hospital, Dhaka, Bangladesh; e Pi Research Consultancy Center, Dhaka, Bangladesh

**Keywords:** Rohingya refugee, Forcibly Displaced Myanmar Nationals, Health problems

## Abstract

**Background:**

Forcibly Displaced Myanmar Nationals (FDMNs) or Rohingya refugees are one of the vulnerable groups suffering from different kinds of health problems but have been less reported yet. Therefore, the study was designed to delineate the health problems among FDMNs admitted to Cox's Bazar Medical College Hospital.

**Methods:**

This hospital-based cross-sectional study was conducted at the Medicine ward, Cox's Bazar Medical College Hospital, for a six-month period following approval. Rohingya refugees who were admitted during the study period were approached for inclusion. Informed written consent was ensured prior to participation. A structured questionnaire was used during data collection. Collected information was recorded in case record form. A total of 290 subjects were interviewed. Analysis was performed using the statistical package for social science (SPSS) version 20.

**Results:**

The mean age of the participants was 48.76 ± 18.67 years (range: 16–91), with a clear male predominance (60.7%). Family size ranged 6–8. All of the participants reported at least one of the illnesses. Of all, 29.66% patients had disease of the respiratory system, and 26.9% had disease of the gastrointestinal and hepatobiliary system. Accidental injury or injury due to electrocution or thin falls or snake bites was present in 10.4% of the cases. Among the single most common diseases, COPD (20%) was the most frequently observed, and the rest of them were chronic liver disease (13.1%), pulmonary TB (5.5%), ischemic stroke (5.5%), CAP (4.1%), acute coronary syndrome (3.4%), thalassaemia (3.4%) and hepatocellular carcinoma (3.4%). Among the top 6 diagnosed diseases, PTB was more common in elderly individuals (*p* = 0.29). The disease pattern was similar across the sexes among the refugees except community acquisition pneumonia (CAP), which was commonly observed among males (*p* = .004). Considering different age groups, genitourinary problems were more common in males aged >60 years, and rheumatology and musculoskeletal problems were equally affected in females aged between 40 and 60 years.

**Conclusion:**

COPD, CLD and CAP were the most prevalent diseases in FDMN patients who attended the Medicine ward of Cox's Bazar Medical College Hospital. Further exploration is warranted before any policy making and comprehensive plan.

## Introduction

Forcibly Displaced Myanmar Nationals (FDMNs), commonly known as Rohingyas, are an ethnic, linguistic and religious minority group of northern Rakhine state (NRS) of Myanmar (Azad and Jasmin, 2013). Clashes and conflicts resulted in displacement in bordering areas of Bangladesh (Health Sector Bulletin, 2017). Although it is not a new phenomenon in the world, approximately 71 million people have been displaced globally because of persecution, conflicts, environmental and other disasters, and among them, 25.9 million are refugees (Bolon et al., 2020). In 1991–1992, more than 250,000 Rohingya refugees fled persecution in the Union of Myanmar and arrived in Bangladesh (Al Masud et al., 2017). More recently, in August 2017, a large displacement occurred, and an estimated 624,000 people fled from Rakhine State to Bangladesh, increasing the total Rohingya population by over 9,00,000 (only in Cox's bazar district) (Chan et al., 2018).

Despite these reasons, this vulnerable population lives in temporary camps and is completely dependent on outside support from the United Nations (UN), the Government of Bangladesh (GOB) and numerous nongovernmental organizations (NGOs) (Idris, 2017). Despite the collaborative assistance of different national and multinational organizations, the overall general health status of refugees is scarcely reported (Inter Sector Coordination Group 2019). They suffered from different kinds of communicable and noncommunicable diseases. Accidents and/or injuries are also common in their camps (Idris, 2017; Holland et al., 2002). Lack of provision of adequate food, water, shelter, sanitation, and the apparently very low level of immunization, creating a perfect storm for the public health situation, were thought to be predictors of their health problems (Idris, 2017; Wijnroks et al., 1993). More recently, a diphtheria outbreak resulted in 38 deaths, and more than 5800 suspected cases of diphtheria were reported as of February 2018. There have also been reports on respiratory problems and skin diseases among refugees, with 10,846 and 3422 cases, respectively (Islam and Nuzhath, 2018).

There are approximately 124 national and international health partners providing services through 169 health facilities (including 7 hospitals) (Health Sector Bulletin, 2017). Approximately 1.2 million people are estimated to be in need of health assistance, including both newly arriving individuals and their host communities (IOM, 2018; World Health Organization, 2017). Irrespective of all health measures, no comprehensive reports have been observed to identify the health problems of this vulnerable population. However, understanding their health problems and proper strategic action plans are required to address the issue both by Govt. and Internationally. For this reason, the study was planned to assess health problems among FDMNs attending the medicine department of Cox's Bazar Medical College Hospital, Bangladesh.

## Materials and methods

### Study design, study site and selection of the patients

This hospital-based cross-sectional study was conducted in the Medicine ward, Cox's Bazar Medical College Hospital, from February 2018 to July 2018. Formal ethical approval was sought prior to conducting the study. Cox's Bazar Medical College Hospital is a tertiary care hospital in Cox's Bazar, Bangladesh, which received patients from around Cox's Bazar district irrespective of the social context of the local and FDMN people. Patients were primarily screened by local doctor (s) at respected refugee camp located in cox's Bazar and referred to cox's Bazar medical college for further evaluation and management. The FDMN patients admitted to the medicine ward suffering from any health conditions were primarily targeted for the study population. FDMN patients aged ≥18 years, admitted to the Medicine ward of Cox's Bazar Medical College Hospital and willing to participate in the study were included in the study. The study was intended to report the disease patterns among FDMN patients. FDMNs who were under aged, pregnant women and not willing to participate were excluded from the study. Moreover, a total of 63 patients were excluded from this study for failing to reach a final diagnosis, mostly due to attrition or non-attendance of the patients (*n* = 32), referral to higher center (*n* = 13) and horizontal referral to other departments of the hospital- Surgery (*n* = 7), Obstetrics and Gynecology (*n* = 11).

### Data collection methods

Data were collected either from patients or their attendants through direct interviews by a semi-structured questionnaire. In all cases, informed written consent was ensured before participation. A preformed questionnaire was used for data collection. The questionnaire consists of four parts: (i) a brief introduction & consent statement, (ii) demographic profile, and (iii) detailed history along with clinical and radiological information of the participants and confirmed diagnosis of the patients. Initially, pretesting of the questionnaire was performed among 10 random participants, and the experience of the piloting was used to make a final adjustment before the final assessment. Hence, a total of 290 interview notes were finally included in the study. The date of disease onset was defined as the day when the symptom was noticed. The clinical parameters included age, sex, family member, time and place from illness onset to hospital admission, comorbidities (systemic hypertension, diabetes mellitus, heart disease, chronic obstructive pulmonary disease, etc.), symptoms, and clinical signs were collected through the questionnaire and were evaluated by trained physicians. General and systematic physical examinations were performed in all patients, including necessary investigations. Data on patient's previous medical records (if available) were also checked.

### Final diagnosis

Diabetes mellitus and hypoglycaemic coma were diagnosed according to ADA (American Diabetic Association) guideline 2018. Along with clinical manifestations, examination findings and relevant blood investigations, cardiac diseases were diagnosed by ECG and echocardiogram (if necessary), respiratory diseases were diagnosed by chest X-ray, gastro-intestinal and urogenital diseases were diagnosed by ultrasonography and computed tomography scan (if necessary). Mental health was assessed by an expert psychiatrist. Disease conditions were classified according to ICD-10 (International classification of diseases-10) classification. All systematic diseases, infectious diseases and accidental diseases were included in this study. All collected data were recorded in a structured case record form and later accumulated and compiled.

### Ethical statement

All procedures performed in studies involving human participants were in accordance with the ethical standards of the institutional (Cox's Bazar Medical College Hospital) and with the 1964 Helsinki declaration and its later amendments or comparable ethical standards. For this type of study, formal consent was ensured. Ethical measures were taken throughout the study period to maintain a high standard of confidentiality and anonymity of the participants. Patients did not bear any costs, as study center is a government hospital, treatment cost was free for every visit and essential medicines. However, the costs of investigations and medications which were out of government facilities, as well as out-of-pocket expenses, were bore by non-government organizations (NGOs) of Rohingya refugee camp.

### Data acquisition and statistical analysis

All of the collected data were entered into a spreadsheet of the statistical software and analyzed with SPSS 20. Descriptive statistics were used during analysis, where continuous variables were expressed as the mean±standard deviation and categorical variables were expressed as count (percentage). To determine the association, the chi-square test was used. All results were analyzed with 95% CIs, and a *p* value <0.05 was considered statistically significant.

## Results

We collected data from 290 individuals who were admitted with various health problems. The mean age of the participants was 48.76 ± 18.67 years (range: 16–91), and the majority were aged >60 years (*n* = 105; 36.2%). Of all, 60.7% were male, and the majority had family members ranging from 6 to 8 (86.2%). Approximately 67% had a previous history of smoking habits. More are described in Table 1.

**Table 1:** Sociodemographic profile of the participants (*n* = 290).

Variables	*n* (%)
Age (years)	48.76 ± 18.67
<40	103 (35.5)
40–60	82 (28.3)
>60	105 (36.2)
Gender	
Male	175 (60.7)
Female	114 (39.3)
Family member	
Below 6	20 (6.9)
6 to 8	250 (86.2)
Above 8	20 (6.9)
Personal habit	
Smoking habit	194 (66.9)
Comorbidities	
DM	16 (5.5)
HTN	22 (7.6)

DM=Diabetes mellitus, HTN= Hypertension

All of the participants had at least one of the illnesses of interest. Of the surveyed respondents, 29.66% individuals had disease of the respiratory system, and 26.9% had disease of the gastrointestinal disease and hepatobiliary system. COPD (20%) ranked at the top of the disease identified in this study. More are described in Table 2.

**Table 2:** Health problems of the respondents (*n* = 290).

Diseases*	*n* (%)
Cardiovascular system	**24 (8.28)**
*CCF*	2 (0.7)
*ICM*	2 (0.7)
*Acute coronary syndrome*	10 (3.4)
*CRHD*	2 (0.7)
*Acute MI*	8 (2.8)
Respiratory system	**86 (29.66)**
*COPD*	58 (20)
*Pulmonary TB*	16 (5.5)
*CAP*	12 (4.1)
GIT and HBS	**78 (26.9)**
*Acute pancreatitis*	6 (2.1)
*Sub-Acute Intestinal Obstruction*	4 (1.4)
*CLD*	38 (13.1)
*Acute hepatitis*	8 (2.8)
*Chronic hepatitis*	2 (0.7)
*CHC infection*	6 (2.1)
*CHB infection*	4 (1.4)
*HBV carrier*	6 (2.1)
*PUD*	2 (0.7)
*Portal Hypertension*	2 (0.7)
Nervous system	**20 (6.9)**
*Acute confusion state*	2 (0.7)
*Ischemic Stroke*	16 (5.5)
*MND*	2 (0.7)
Endocrine system	**8 (2.8)**
*Diabetic peripheral neuropathy*	2 (0.7)
*DM*	4 (1.4)
*Hypoglycemic Coma*	2 (0.7)
Genito-urinary system	**12 (4.2)**
*PKD*	4 (1.4)
*AKI*	2 (0.7)
*BPH*	2 (0.7)
*CKD*	2 (0.7)
*Nephrotic Syndrome*	2 (0.7)
Infection	**10 (3.4)**
*Encephalitis*	2 (0.7)
*Enteric Fever*	2 (0.7)
*Liver Abscess*	2 (0.7)
*Acute pyelonephritis*	2 (0.7)
*Complicated UTI*	2 (0.7)
Malignancy	**20 (6.8)**
*Stomach cancer*	2 (0.7)
*HCC*	10 (3.4)
*Lung cancer*	6 (2.1)
*CLL*	2 (0.7)
Hematology	**10 (3.4)**
*Thalassemia*	10 (3.4)
Psychiatric disorder	4 (1.4)
*Schizophrenia*	4 (1.4)
Rheumatology and musculoskeletal	**8 (2.8)**
*RA*	2 (0.7)
*SLE*	2 (0.7)
*Cervical myelopathy*	2 (0.7)
*Lumbago Sciatica*	2 (0.7)
Accident and injury	**10 (3.4)**
*Electrocution*	4 (1.4)
*Thunder fall*	4 (1.4)
*Snake Bite*	2 (0.7)

HTN-Hypertension, DM=Diabetes Mellitus, COPD= Chronic Obstructive Pulmonary Disease, CLD= Chronic Liver Disease, CAP= Community Acquired Pneumonia, HCC= Hepatocellular Carcinoma, HBV= Hepatitis B virus, PKD= Polycystic Kidney Disease, RA= Rheumatoid Arthritis, SLE= Systemic lupus Erythematosus, AKI= Acute Kidney Injury, BPH= Benign Prostatic Hyperplasia, CKD= Chronic Kidney Disease, UTI= Urinary Tract Infection, CCF= Congestive Cardiac Failure, CRHD=Chronic Rheumatic Heart Disease, TB= Tuberculosis, ICM= Ischemic Cardio Myopathy, MND= Motor Neuron Disease, PUD= Peptic Ulcer Disease. MI= Myocardial Infarction

Among the top 6 reported diseases, PTB was linked with age difference and was more common in elderly individuals (*p* = 0.29). In contrast, diseases of the rheumatology and musculoskeletal system have a propensity to occur in people aged 40–60 years (*p* < 0.001). More are illustrated in Table 3.

⁎ Multiple response

**Table 3:** Differences in the distribution of diseases according to age group (*N* = 290).

Diseases⁎⁎	Age group			*p* value*
	<40 years (*N* = 103)	40 to 60 years (*N* = 82)	>60 years (*N* = 105)	
	*n* (%)	*n* (%)	*n* (%)	
Top 6 Diseases				
*COPD*	25 (24.3)	14 (17.1)	19 (18.1)	0.396
*CLD*	20 (19.4)	8 (9.8)	10 (9.5)	0.061
*CAP*	2 (1.9)	6 (7.3)	4 (3.8)	0.185
*PTB*	2 (1.9)	4 (4.9)	11 (10.5)	0.029
*Acute coronary syndrome*	4 (3.9)	2 (2.4)	4 (3.8)	0.839
*Ischemic Stroke*	4 (3.9)	4 (4.9)	8 (7.6)	0.477
Systems				
*Cardiovascular*	10 (9.7)	4 (4.9)	10 (9.5)	0.419
*Respiratory*	29 (28.2)	24 (29.3)	33 (31.4)	0.871
*GIT and HBS*	33 (32)	18 (22)	27 (25.7)	0.289
*Nervous*	6 (5.8)	6 (7.3)	8 (7.6)	0.864
*Endocrine*	3 (2.9)	2 (2.4)	7 (6.7)	0.262
*Genito-urinary*	2 (1.9)	0	6 (5.7)	0.050
*Infection*	2 (1.9)	4 (4.9)	4 (3.8)	0.536
*Malignancy*	12 (11.7)	4 (4.9)	4 (3.8)	0.058
*Hematology*	4 (3.9)	4 (4.9)	2 (1.9)	0.519
*Psychiatric disorder*	0	2 (2.4)	2 (1.9)	0.312
*Rheumatology and musculoskeletal*	0	8 (9.8)	0	<0.01
Others				
*Accident and injury*	2 (1.9)	6 (7.3)	2 (1.9)	0.077

COPD= Chronic Obstructive Pulmonary Disease, CLD= Chronic Liver Disease, CAP= Community Acquired Pneumonia, PTB=Pulmonary tuberculosis, GIT-Gastrointestinal tract, HBS=Hepatobiliary system

The disease pattern was similar across the sexes among the refugees except community acquisition pneumonia (CAP), which was commonly observed among males (*p* = .004) **(**Table 4**).**

⁎ *p* value was determined by Chi-square test. Bonferroni correction found that column proportion of age group categories do not differ significantly from each other at the 0.05 level.

⁎⁎ Multiple response

**Table 4:** Pattern of diseases according to sex difference (*n* = 290).

Diseases⁎⁎	Gender		*p* value*
	Male(*n* = 176)	Female(*n* = 114)	
	n (%)	n (%)	
**Top 6 Diseases**			
COPD	29 (16.5)	29 (25.4)	0.062
CLD	21 (11.9)	17 (14.9)	0.463
CAP	12 (6.8)	0	0.004
PTB	9 (5.1)	8 (7)	0.500
Acute coronary syndrome	7 (4)	3 (2.6)	0.540
Ischemic Stroke	8 (4.5)	8 (7)	0.368
**System**			
Cardiovascular	19 (10.8)	5 (4.4)	0.053
Respiratory	49 (27.8)	37 (32.5)	0.401
GIT and HBS	49 (27.8)	29 (25.4)	0.652
Nervous	11 (6.3)	9 (7.9)	0.589
Endocrine	6 (3.4)	6 (5.3)	0.439
Genito-urinary	6 (3.4)	2 (1.8)	0.401
Infection	6 (3.4)	4 (3.5)	0.964
Malignancy	14 (8)	6 (5.3)	0.377
Hematology	5 (2.8)	5 (4.4)	0.481
Psychiatric disorder	2 (1.1)	2 (1.8)	0.659
Rheumatology and musculoskeletal	4 (2.3)	4 (3.5)	0.530
Accident and injury	5 (2.8)	5 (4.4)	0.481

COPD= Chronic Obstructive Pulmonary Disease, CLD= Chronic Liver Disease, CAP= Community Acquired Pneumonia, PTB=Pulmonary tuberculosis, GIT-Gastrointestinal tract, HBS=Hepatobiliary system

Man with higher chronological age (>60 y) suffer more from genito-urinary problems, while rheumatology and musculoskeletal problems are commonly encountered in people aged 40-60 years irrespective of gender. For more details see Table 5**.**

⁎ *p* value was determined by Chi-square test

⁎⁎ Multiple response

**Table 5:** Pattern of diseases with respect to age and sex (*n* = 290).

Diseases⁎⁎	Male			*p* value*	Female			*p* value*
	<40 years (*n* = 64)	40 to 60 years (*n* = 50)	>60 years(*n* = 62)		<40years (*n* = 39)	40 to 60 years (*n* = 32)	>60 years (*n* = 43)	
	n (%)	n (%)	n (%)		n (%)	n (%)	n (%)	
Top 6 Diseases								
COPD	13 (20.3)	7 (14)	9 (14.5)	0.583	12 (30.8)	7(21.9)	10 (23.3)	0.636
CLD	12 (18.8)	4 (8)	5 (8.1)	0.108	8 (20.5)	4 (12.5)	5 (11.6)	0.478
CAP	2 (3.1)	6 (12)	4 (6.5)	0.174	0	0	0	
PTB	1 (1.6)	2 (4)	6 (9.7)	0.108	1 (2.6)	2 (6.3)	5 (11.6)	0.270
Acute coronary syndrome	3 (4.7)	1 (2)	3 (4.8)	0.699	1 (2.6)	1 (3.1)	1 (2.3)	0.977
Ischemic Stroke	2 (3.1)	2 (4)	4 (6.5)	0.653	2 (5.1)	2 (6.3)	4 (9.3)	0.746
Systems								
Cardiovascular	7 (10.9)	3 (6)	9 (14.5)	0.352	3 (7.7)	1 (3.1)	1 (2.3)	0.455
Respiratory	16 (25)	15 (30)	18 (29)	0.812	13 (33.3)	9 (28.1)	15 (34.9)	0.817
GIT and HBS	23 (35.9)	11 (22)	15 (24.2)	0.187	10 (25.6)	7 (21.9)	12 (27.9)	0.838
Nervous	3 (4.7)	4 (8)	4 (6.5)	0.766	3 (7.7)	2 (6.3)	4 (9.3)	0.888
Endocrine	2 (3.1)	1 (2)	3 (4.8)	0.704	1 (2.6)	1 (3.1)	4 (9.3)	0.321
Genito-urinary	1 (1.6)	0	5 (8.1)	**0.039**	1 (2.6)	0	1 (2.3)	0.670
Infection	1 (1.6)	3 (6)	2 (3.2)	0.430	1 (2.6)	1 (3.1)	2 (4.7)	0.868
Malignancy	8 (12.5)	3 (6)	3 (4.8)	0.236	4 (10.3)	1 (3.1)	1 (2.3)	0.225
Hematology	2 (3.1)	2 (4)	1 (1.6)	0.741	2 (5.1)	2 (6.3)	1 (2.3)	0.687
Psychiatric disorder	0	1 (2)	1 (1.6)	0.551	0	1 (3.1)	1 (2.3)	0.569
Rheumatology and musculoskeletal	0	4 (8)	0	**0.006**	0	4 (12.5)	0	**0.006**
Accident and injury	1 (1.6)	3 (6)	1 (1.6)	0.283	1 (2.6)	3 (9.4)	1 (2.3)	0.267

COPD= Chronic Obstructive Pulmonary Disease, CLD= Chronic Liver Disease, CAP= Community Acquired Pneumonia, PTB=Pulmonary tuberculosis, GIT-Gastrointestinal tract, HBS=Hepatobiliary system

⁎ *p* value was determined by Chi-square test. Bonferroni correction found that column proportion of age group categories do not differ significantly from each other at the 0.05 level.

⁎⁎ Multiple response

## Discussion

Forcibly Displaced Myanmar Nationals (FDMNs) to Bangladesh are currently the world's largest and most densely populated refugee population (Khan et al., 2020), causing human suffering on a catastrophic scale. Due to the increasing number of Rohingya refugees and their congested living conditions in camps, there has been an overwhelming increase in their health risks. Against this backdrop, it is important to ensure health services for the Rohingya population, and to do so, knowing about their current health status is imperative because without this information, equal and equitable health service provision, as well as appropriate resource allocation, is not possible. In addition, failure to provide adequate health service and thus to maintain the sound health of Rohingya refugees might adversely affect the health status of Bangladeshi people. Therefore, we conducted this study with the aim of understanding the current health status of FDMNs in Bangladesh. Total 290 patients were included. The majority of the patients were younger than 60 years, with a mean age of 49 years (range: 16–91). A study by Al Masud et al. (2017) on the health problems and health care seeking behavior of Rohingya refugees found an almost similar age distribution, with a mean of 46 years, and the majority of refugees were 19–59 years of age. However, several studies found different patterns of age distribution in our findings, which might be due to the larger sample size and inclusion of child groups in the population (Islam and Nuzhath, 2018; Joarder et al., 2020).

In this study, we found a male predominance (60.7%) compared to females (39.3%). This result is incompatible with other studies where female sex was predominantly higher than male sex (Al Masud et al., 2017; Islam and Nuzhath; Joarder et al., 2020). However, the reason of male preponderance in this study is probably the effect of the study site, as this study was confined to the medicine ward in Cox's bazar Medical College Hospital, where gynae and obstetrical services and surgical services were not provided. Hence, data on sexual violence and female reproductive diseases were not available in our study. Moreover, as this study excluded child groups, a significant portion of females, especially children, were not included in these findings.

In our study, the majority of patients suffered from respiratory system diseases, followed in decreasing order by GIT and hepato-biliary system, cardio-vascular system, nervous system, malignancy and genito-urinary system. Among the most prevalent diseases, COPD was ranked on top, followed by chronic liver disease, pulmonary TB, ischemic stroke, CAP, acute coronary syndrome, thalassaemia and hepatocellular carcinoma. The high prevalence of COPD in this study might be due to the fact that about 2/3rd of our study population are smoker, as smoking the main cause of COPD (Forey et al., 2011). In addition, we found that 7.6% of patients had HTN and 5.5% had a history of DM. A similar study performed by Al Masud et al. (2017) found that urinary tract infection (UTI) was the leading individual health problem, followed by hypertension, respiratory tract infection, nutrition deficiency and diabetes mellitus. The overall scenario was slightly different from our study, as that study was conducted at a primary care center and our study was conducted in a tertiary care setting. However, there is a scarcity of evidence on disease patterns among Rohingya refugees in Bangladesh to compare our study findings, although the high prevalence of noncommunicable diseases (NCDs) such as COPD, cardiovascular diseases, chronic liver diseases, diabetes, hypertension, and malignancy among adults in humanitarian settings across the globe is comparable to our findings (Duckles et al., 2018; Lee et al., 2012; Dookeran et al., 2010, Kinzie et al., 2008). These findings might be explained by the high exposure of the refugee population to different behavioral and environmental risk factors for developing NCDs (Dharod et al., 2013). However, prevention and early detection of NCDs are undoubtedly more cost-effective than managing the later stages of disease, as increasing severity and associated complications of NCDs concurrently increase morbidity and mortality for refugees (Yun et al., 2012).

This study found that community acquired pneumonia was more common in males than females, which was also supported by previous literatures (Marini et al., 2018; Barbagelata et al., 2020). The underlying mechanisms driving the observed differences between men and women are not discernible from our available data. Nonetheless, sex-specific preventive strategies to reduce the risk of these health complications may be one strategy to improve health of Rohingya refugees.

Doctors of the world have found a high prevalence of mental health problems and psychological distress in migrant and refugee populations, including posttraumatic stress disorder, depression, anxiety, sleep disturbance, substance misuse and somatization (Daynes, 2016), as a consequence of violence, and migration-related factors, such as adjusting to a different environment in a new country (Joarder et al., 2020). In this study, we found only 4 patients (1.4%) with psychiatric disorders, specifically schizophrenia, which might be because only referred cases of mental disorders were admitted to the medicine ward and included as our study patients.

This study suggests that an organized primary health care service, specially designed for communicable and non-communicable disease prevention and management is necessary to improve health condition of FDMNs. Strong primary health care – organized in multi-disciplinary teams and with innovative roles for health professionals, integrated with community health services, equipped with digital technology, and working with well-designed incentives – may help to deliver a successful health system response in FDMNs. Primary health care and hospital services should work together through effective team-based health care. Therefore, collaboration must include governmental agencies, businesses, and community-based organizations who are best positioned to create policies and practices that promote healthy environments.

## Limitations

The study has several limitations, including a small sample size, and the study was only confined to cases observed in the medicine department. Hence, it may not be reflective of actual health problems among the Rohingya refugees lived in Bangladesh.

## Conclusion

The Rohingya population mostly suffers from COPD, CLD and CAP. With few exceptions, the disease pattern was similar across different age groups and genders. Hence, preventive actions should be incorporated to minimise respiratory and gastrointestinal health problems in Rohingya refugees.

## Supplementary materials

Available on request

## Ethical consideration

Ethical measures were taken throughout the study period to maintain a high standard of confidentiality and anonymity of the participants. Formal approval was taken from the ethical review committee of Cox's Bazar Medical College.

## Consent for publication

The author agreed to publish the article by written consent.

## CRediT authorship contribution statement

**Mohammad Ismail:** Conceptualization, Formal analysis, Investigation, Methodology, Resources, Supervision, Writing – original draft, Writing – review & editing. **Mohammad Farhad Hussain:** Conceptualization, Investigation, Methodology, Resources, Writing – review & editing. **Mohammad Abdullah al Hasan:** Conceptualization, Investigation, Investigation, Resources, Writing – review & editing. **AHM Mustafa Kamal:** Conceptualization, Investigation, Methodology, Resources, Writing – review & editing. **Monjur Rahman:** Conceptualization, Formal analysis, Supervision, Writing – original draft. **Mohammad Jahid Hasan:** Conceptualization, Formal analysis, Methodology, Resources, Supervision, Writing – original draft, Writing – review & editing.

## Declaration of Competing Interest

None.
